# Sweet cherry TCP gene family analysis reveals potential functions of *PavTCP1*, *PavTCP2* and *PavTCP3* in fruit light responses

**DOI:** 10.1186/s12864-023-09923-z

**Published:** 2024-01-02

**Authors:** Chaoqun Chen, Yao Zhang, Yuanfei Chen, Hongxu Chen, Ronggao Gong

**Affiliations:** College of Horticulture, Sichuan Agricultural University, Chengdu, 6111130 China

**Keywords:** Sweet cherry, Genome-wide, Anthocyanin, miRNA, Bagging

## Abstract

**Background:**

TCP proteins are plant specific transcription factors that play important roles in plant growth and development. Despite the known significance of these transcription factors in general plant development, their specific role in fruit growth remains largely uncharted. Therefore, this study explores the potential role of TCP transcription factors in the growth and development of sweet cherry fruits.

**Results:**

Thirteen members of the *PavTCP* family were identified within the sweet cherry plant, with two, *PavTCP1* and *PavTCP4*, found to contain potential target sites for Pav-miR159, Pav-miR139a, and Pav-miR139b-3p. Analyses of cis-acting elements and *Arabidopsis* homology prediction analyses that the *PavTCP* family comprises many light-responsive elements. Homologs of *PavTCP1* and *PavTCP3* in Arabidopsis TCP proteins were found to be crucial to light responses. Shading experiments showed distinct correlation patterns between *PavTCP1*, *2*, and *3* and total anthocyanins, soluble sugars, and soluble solids in sweet cherry fruits. These observations suggest that these genes may contribute significantly to sweet cherry light responses. In particular, *PavTCP1* could play a key role, potentially mediated through Pav-miR159, Pav-miR139a, and Pav-miR139b-3p.

**Conclusion:**

This study is the first to unveil the potential function of *TCP* transcription factors in the light responses of sweet cherry fruits, paving the way for future investigations into the role of this transcription factor family in plant fruit development.

**Supplementary Information:**

The online version contains supplementary material available at 10.1186/s12864-023-09923-z.

## Background

Transcription factors are proteins that regulate gene transcription and expression by binding to gene-specific sequences, thereby influencing cell biological activity [1]. The TCP family, a plant-specific family of transcription factors, plays pivotal roles in numerous facets of plant growth and development [2, 3]. First reported in 1999 [4], the family’s name derives from the initials of four originally characterized members: teosinte branched1 (TB1) in maize [5], CYCLOIDEA (CYC) in *Antirrhinum majus* [6], and proliferating cell factors 1 and 2 in rice (*Oryza sativa* L.) (PCF1 and PCF2) [7]. Every *TCP* family member contains a highly conserved non-canonical basic helix-loop-helix (bHLH) motif known as the TCP domain. Comprising 59 amino acids, the TCP domain regulates DNA binding, nuclear targeting, and protein interactions [4]. Based on TCP domain homology, the *TCP* family is divided into two subfamilies: class I (TCP-P subfamily) and class II (TCP-C subfamily). The latter is further divided into CINCINNATA (CIN) and angiosperm-specific CYC/TB1 [8]. The primary distinction between these two classes is a four amino acid deletion in the basic region of the TCP structural domain in class I. Moreover, class II families usually feature a unique arginine-rich R structural domain, predominantly found in the CYC/TB1 branch [9]. Despite their distinct DNA-binding site sequences (GGNCCCAC for class I and GTGGNCCC for class II), these sequences overlap and share a common core (GGNCC) [10].

Class I TCPs generally promote cell proliferation and plant growth [11], while class II TCPs mainly inhibit cell differentiation and plant growth [3]. The TCP transcription factor family has been identified in a diverse array of plants, but unlike other transcription factor families, such as MYB (V-myb avian myeloblastosis viral), WRKY (WRKY proteins contain one or two conserved WRKYGQK motifs at the N-terminus and a typical zinc-finger motif (C2H2 and C2HC) at the C-terminus), and NAC (The name of the NAC gene family is composed of the initials NAM (No apical meristem from *Petunia hybrida*), AF1/2 (transcriptional activator 1/2 from *Arabidopsis thaliana*), and CUC2 (cup-shaped cotyledon from *Arabidopsis thaliana*) transcription factors.), the TCP family usually comprises fewer members, with 24 in *Arabidopsis thaliana* [11], 32 in morning glory (*Catharanthus roseus*) [12], 27 in watermelon (*Citrullus lanatus*) [13], 26 in Chinese cabbage (*Brassica rapa*) [14], 30 in tomato (*Solanum lycopersicum*) [15], 20 in citrus (*Citrus*) [16], 26 in bitter buckwheat (*Fagopyrum esculentum*) [17], 40 in moso bamboo (*Phyllostachys pubescens*) [18], 18 in grape (*Vitis vinifera*) [19], and 62 in blueberry (*Vaccinium corymbosum*) [20]. The functions of the *TCP* family have been revealed over time, from initially preventing axillary bud tip growth [5] and controlling floral symmetry [6] to addressing abiotic stresses [14, 16, 17], biotic stresses [21], plant height [13], fruit maturation [15], anthocyanin accumulation [9, 22], hormonal pathways [19], and bud dormancy [20]. Earlier studies have demonstrated that *SlTCP12*, *SlTCP25*, and *SlTCP18* (SI stands for *Solanum lycopersicum* L. acronym.), which are preferentially expressed in tomato fruits, are regulated by ripening inhibitor, colorless non-ripening, and APETALA2a proteins. This suggests that *TCP* transcription factors contribute to the development or ripening of tomato fruits, marking the first time the *TCP* transcription factor family has been linked to the development of fleshy fruit [15].

MicroRNAs (miRNAs) are small non-coding RNAs that typically regulate gene expression by inhibiting messenger RNA (mRNA) translation or promoting mRNA degradation at the post-transcriptional level [23]. Numerous studies have revealed that miRNAs mediate various biological functions in different plants. For example, in *Arabidopsis thaliana*, MiR858 manages the regulation of flavonoid-specific *MYB* transcription factors to control the plant’s resistance to pathogen infection [24]. In rice, Osa-miR1320 targets the *ERF* transcription factor *OsERF096* to govern cold tolerance via JA-mediated signaling [25]. Within the *TCP* transcription factor family, miRNA-mediated regulation exists, with miR319 being the main miRNA family member playing this role. For instance, in switchgrass (*Panicum virgatum* L.), *PvPCF5* (Pv stands for *Panicum virgatum* L. acronym.) binds to miR319 and controls lignin biosynthesis [26]. In watermelon, *TCP* transcription factors can also be regulated by miR319 to enhance fruit salt tolerance [27].

Sweet cherry (*Prunus avium* L.), a member of the Prunus genus in the Rosaceae family, was initially cultivated in the region between northeastern Anatolia, the Caucasus, and the Caspian Sea [28]. It is now grown in most countries with a mild climate worldwide [29]. Due to their bright color, sweet and juicy flavor, and high nutritional value, sweet cherries are highly popular in fruit markets and have significant economic value [30]. They are rich in monosaccharides (glucose, fructose, sucrose, and sorbitol), vitamins, minerals, phenolic compounds (flavonoids and anthocyanosides), and dietary fiber [31], which play a crucial role in reducing cancer risk and combating diseases such as joint pain [32]. While several gene families and functions, such as *HD-ZIP* [33], *MYB* [34], *GST* [35], and *bHLH* [36], have been identified and analyzed in sweet cherry, the role of the *TCP* transcription factor family in the light responses of sweet cherry fruit is not well reported. This study identified 13 PavTCPs (Pav stands for *Prunus avium* L. acronym.) and analyzed the correlation with the physiological indices after shading. The results showed that the expression of three transcription factors (*PavTCP1, PavTCP2*, and *PavTCP3*) have high relevance with total anthocyanin, soluble sugar, and soluble solid contents, which indicated that the three transcription factors may be the potential regulators of fruit quality.

## Results

### Chromosome localization and phylogenetic analysis

Based on the HMMER search results, a total of 13 *TCP* genes with typical TCP conserved structural domains were obtained and annotated as *TCP1-TCP13*. The physical information of all identified *TCP* genes in sweet cherry is presented in Table 1. There are many differences between each PavTCP in the amino acids, molecular weight, and theoretical PI. The *PavTCP9* contains the most amino acids at 506, and its molecular weight is 57,580.77. The theoretical PI changes from 5.67 to 9.51. It was similar to most other research that the *TCP* genes located in the nucleus.

**Table 1:** Basic predicted information of PavTCPs

Gene name	Gene ID	Number of amino acids	Molecular weight/daltons	Theoretical pI	Group	Subcellular localization
PavTCP11	Pav_sc0000220.1_g1650.1.mk	425	44,902.68	7.01	Groupl-PCF	nucleus
PavTCP10	Pav_sc0000877.1_g280.1.mk	389	41,579.99	9.36	Groupl-PCF	nucleus
PavTCP13	Pav_sc0000555.1_g160.1.mk	268	28,608.93	9.51	Groupl-PCF	nucleus
PavTCP12	Pav_sc0001080.1_g800.1.mk	308	33,077.77	9.01	Groupl-PCF	nucleus
PavTCP4	Pav_sc0001340.1_g140.1.mk	378	40,993.59	6.36	Groupll-CIN	nucleus
PavTCP5	Pav_sc0002264.1_g280.1.mk	457	50,895.56	6.34	Groupll-CIN	nucleus
PavTCP6	Pav_sc0000037.1_g020.1.mk	292	33,416.72	5.67	Groupll-CIN	nucleus
PavTCP1	Pav_sc0003135.1_g360.1.br	492	53,435.08	7.31	Groupll-CIN	nucleus
PavTCP2	Pav_sc0000358.1_g330.1.mk	388	42,864.89	6.95	Groupll-CIN	nucleus
PavTCP3	Pav_sc0002358.1_g040.1.mk	376	41,438.64	6.54	Groupll-CIN	nucleus
PavTCP7	Pav_sc0003685.1_g070.1.mk	502	55,842.61	8.61	Groupll-CYC/TB1	nucleus
PavTCP9	Pav_sc0000383.1_g850.1.mk	506	57,580.77	6.46	Groupll-CYC/TB2	nucleus
PavTCP8	Pav_sc0000618.1_g400.1.mk	417	46,573.99	8.29	Groupll-CYC/TB3	nucleus

As shown in Fig. S1, a total of nine chromosomes are present in the sweet cherry and the 13 *TCP* genes were unevenly distributed across five chromosomes. No genes were localized on chromosome 2. chromosome 4 had the most distributed genes (4), namely, *PavTCP1*, *PavTCP4, PavTCP5,* and *PavTCP6*. There were 3 genes on each of chromosome 1 (*PavTCP7, PavTCP11, PavTCP13*) and chromosome 5 (*PavTCP2, PavTCP9, PavTCP10*), 2 genes on chromosome 3 (*PavTCP8, PavTCP12*), and only 1 TCP gene (*PavTCP3*) on chromosome 0.

**Fig. 1:**
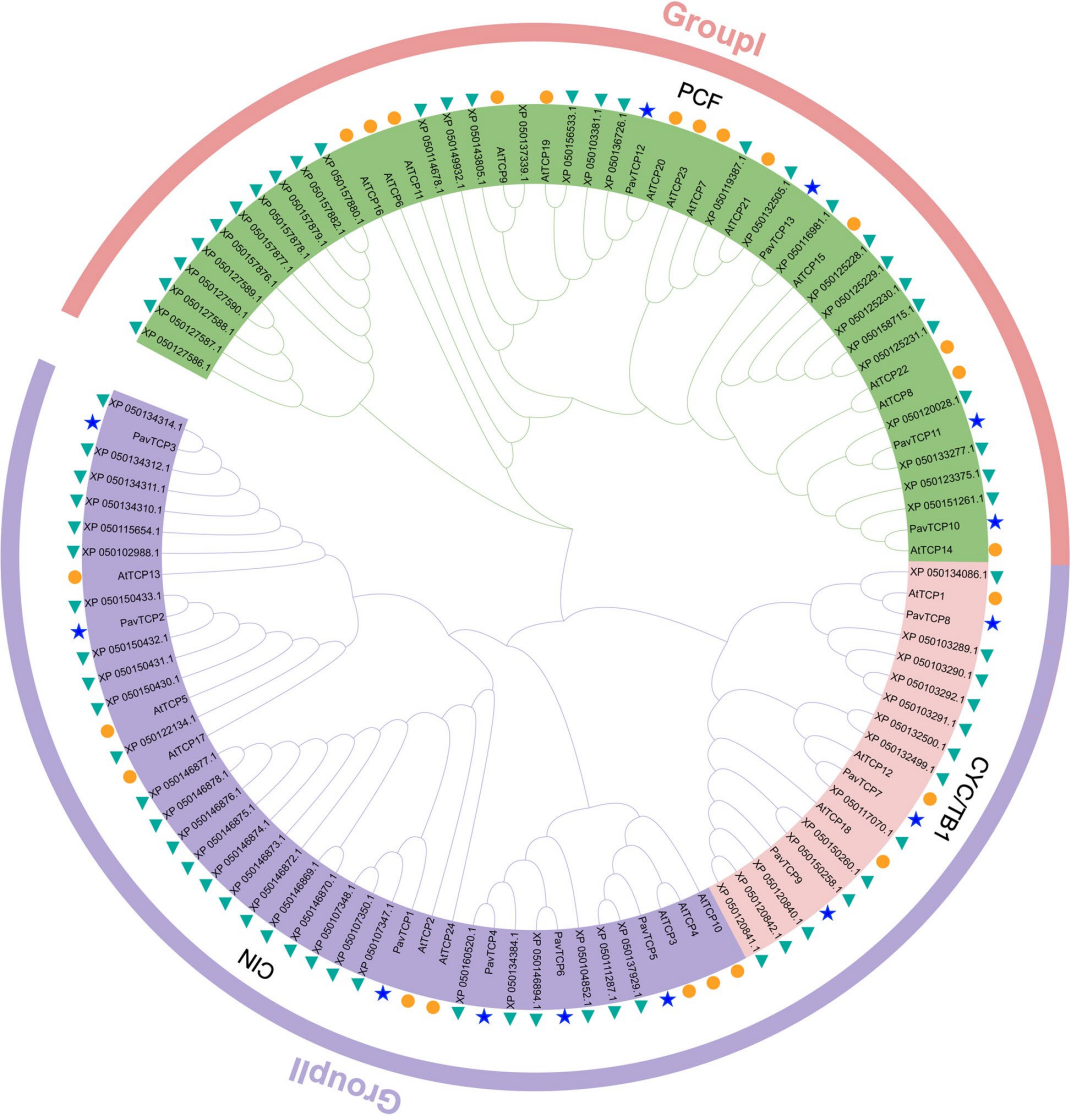
Phylogenetic analysis of TCP proteins in sweet cherry, Arabidopsis, and apple. The asterisks mark the sweet cherry *TCP* gene, the triangles mark the apple *TCP* gene, and the circles mark the Arabidopsis *TCP* gene. PCF, CIN, and CYC/TB1 indicated different subfamilies, respectively

To further establish the evolutionary relationships of the sweet cherry TCP gene family, a phylogenetic tree was constructed using protein sequences of *Arabidopsis thaliana*, apple, and sweet cherry (Fig. 1). Based on multiple sequence alignment of 13 PavTCPs, 24 AtTCPs, and 71 apple TCP proteins, two TCP subfamilies, Group I and Group II, were generated using the MEGA7.1 method. Group I was named PCF, and Group II was further classified into CIN and CYC/TB1. The CYC/TB1-type genes were the least abundant (19) and contained three PavTCPs (*PavTCP7*, *PavTCP8*, *PavTCP9*). The CIN subfamily contained the most PavTCPs (6), with four PavTCPs located on Chr (*PavTCP1*, *PavTCP4*, *PavTCP5, PavTCP6)* all included in this family, in addition to 8 AtTCPs and 29 MdTCPs (Md stands for *Malus domestica* Borkh.) in this subgroup. The PCF subfamily contained 4 PavTCPs (*PavTCP12*, *PavTCP10*, *PavTCP11*, and *PavTCP13*), 29 MdTCPs, and 13 AtTCPs. Unlike sweet cherry, *Arabidopsis thaliana TCP* family members were most distributed in this subgroup. Phylogenetic relationships suggest that some sweet cherry *TCP* gene family members may have undergone species-specific evolutionary processes.

### Collinearity analysis of PavTCPs

**Fig. 2:**
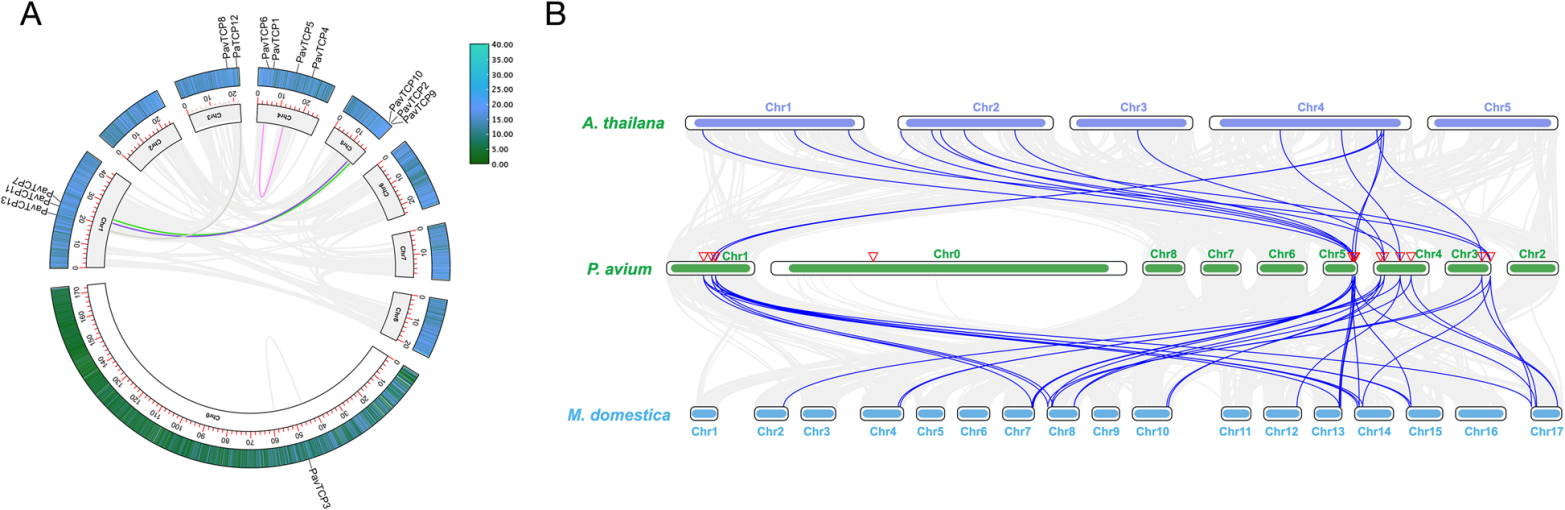
Collinearity analysis. **A** Covariance analysis of all PavTCPs in the sweet cherry genome. The blue to green color indicates the size of the distribution density of the gene on the chromosome. **B** Covariance analysis of the genomes of Arabidopsis, sweet cherry, and apple. Chr denotes chromosome

We further analyzed PavTCPs for intraspecific colinearity (Fig. 2A). The analysis revealed that there were four pairs of duplication events involving eight PavTCPs. Specifically, *PavTCP5* and *PavTCP6* located on chromosome 4 were identified as tandem duplications. Additionally, three pairs of segmental duplications occurred on chromosome 1 (*PavTCP7*) and chromosome 5 (*PavTCP9*), chromosome 1 (*PavTCP11*) and chromosome 5 (*PavTCP10*), and chromosome 1 (*PavTCP13*) and chromosome 3 (*PavTCP12*), with the majority of duplication events occurring on chromosome 1. Gene clusters refer to two or more genes located within 20 kb. We identified four PavTCP gene clusters involving 12 genes. The total duplication rate of PavTCPs was 61.54% (8/13), suggesting that duplication events contributed significantly to the expansion of the PavTCP gene family.

To gain further insights into the phylogenetic relationships of PavTCPs, we carried out an interspecific collinearity analysis using homologous *TCP* gene pairs from Arabidopsis and apple (Fig. 2B). The analysis revealed that a total of 12 *PavTCP* genes were homologous to apple, forming 35 homologous gene pairs. Nine sweet cherry *TCP* genes had homology to those in Arabidopsis, forming 16 homologous gene pairs. Some colinearity relationships were observed only in apple and not in Arabidopsis. This suggests that the collinearity relationship between MdTCPs and PavTCPs was more abundant than that of AtTCPs, indicating that sweet cherry *TCP* genes were more homologous to apple *TCP* genes. Additionally, no collinearity was found between *PavTCP3*, located on sweet cherry chromosome 0, and *MdTCP* and *AtTCP*, suggesting that the evolutionary relationship of this *TCP* gene may be unique in the sweet cherry genome.

### Promoter Cis-acting element analysis

**Fig. 3:**
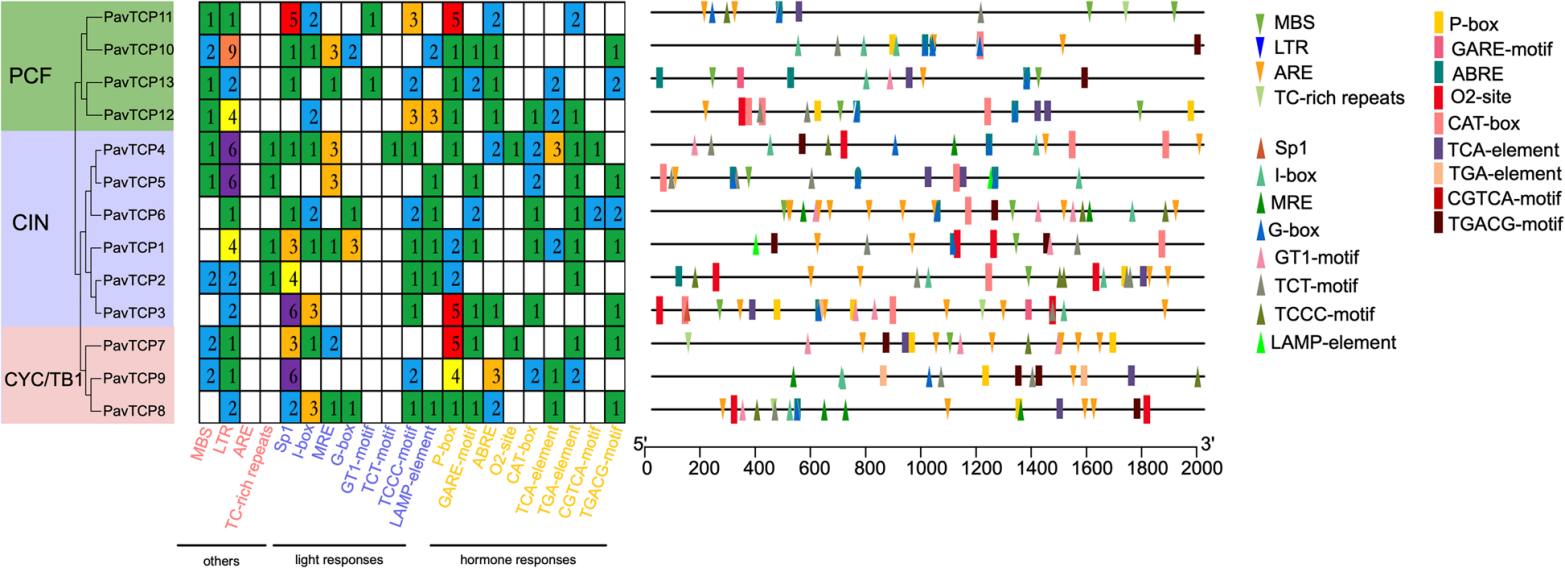
Gene cis-acting element analysis of the promoter. Small squares of different colors indicate the number of cis-acting element contained

To gain a deeper understanding of the regulatory mechanisms of PavTCPs, we extracted the first 2000 bp of a total of 13 gene sequences as promoter region sequences and submitted them to the plantCARE website for cis-acting element prediction (Fig. 3, Table S7). In addition to the common CAAT-box and TATA-box, we also predicted a multitude of other elements impacting plant growth and development and categorized them into light-responsive elements, hormone-responsive elements, and other elements. Among all the cis-acting elements of the 13 genes, hormone-responsive elements were the most abundant. Nevertheless, the elements were not highly specific; only the CGTCA motif was specifically present in the CIN subfamily, with the remaining cis-acting elements of this class being distributed in all subfamilies of the sweet cherry *TCP* gene family. The P-box was the most abundant in the hormone-responsive class of elements of the PavTCPs. Among the light-responsive elements, the TCT motif was specifically present only in *PavTCP4* of the CIN subfamily, and the GT1 motif was only present in *PavTCP11* and *PavTCP13* of the PCF subfamily. Sp1 was the most abundant of the light-responsive class of elements in the PavTCPs, and similar to the P-box, the element constituted a large proportion in the CYC/TB1 subfamily, that it may play a role in regulating plant growth and development in PavTCPs.

### Conserved domains and motif analysis

**Fig. 4:**
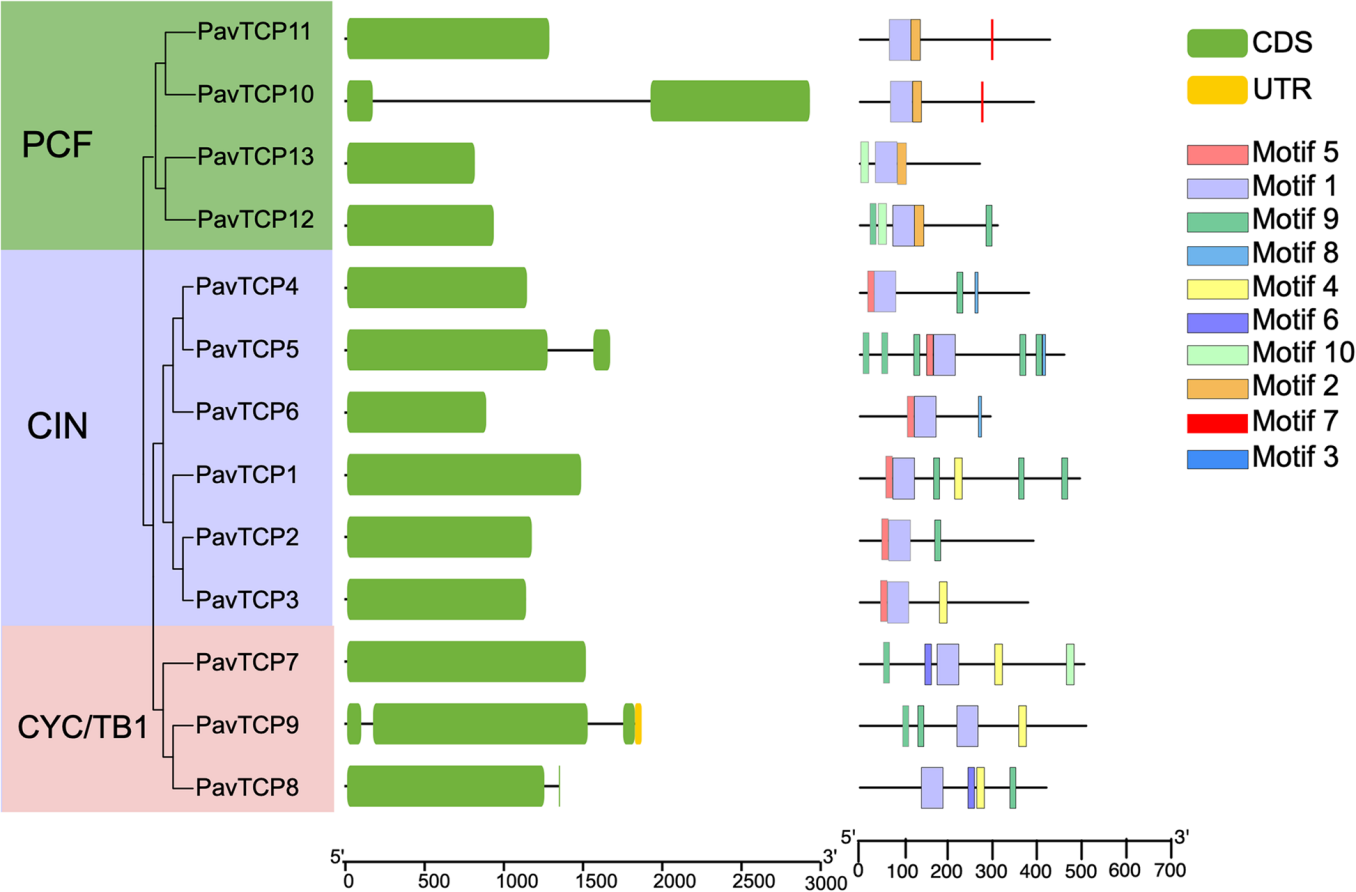
Gene structure and motif position of the *PavTCP* genes

Further analysis of the PavTCP family gene structure revealed that all 13 *TCP* genes in sweet cherry encompassed coding regions (CDS) that varied in number from 1 to 3 (Fig. 4). Specifically, *PavTCP9* in the CYC/TB1 subfamily contained an untranslated region, and this gene had the highest number of CDS (3). Similar to the conserved motif analysis, the quantity and distribution of CDSs and UTRs in the three genes of the CYC/TB1 subfamily were dissimilar, suggesting that the gene structure of this subfamily is not highly conserved. Conversely, all genes in the CIN subfamily, apart from *PavTCP5*, contained only one CDS with similar CDS lengths, signifying that the CIN subfamily has a more conserved structure than the other subfamilies. Nonetheless, overall, the UTR-CDS structure of the sweet cherry *TCP* gene family displayed a high degree of simplicity.

To gain a better understanding of the diversity and conserved structure of sweet cherry PavTCP protein sequences, we analyzed the conserved motifs of PavTCPs using MEME online tool and identified a total of 10 different conserved motifs (Fig. 4, Fig. S2). The results indicated that *PavTCP* members within the same subgroup had similar motif compositions, and all TCP proteins contained Motif1. Motif2 and Motif5 were specific to the PCF and CIN subfamilies and were present in all TCP proteins of these two subfamilies, respectively. Motif7 was specifically present in *PavTCP10* and *PavTCP11* of the PCF subfamily, and these two proteins exhibited the same motif composition and distribution. In addition, Motif8 was specifically present in *PavTCP4*, *PavTCP5*, and *PavTCP6* of the CIN subfamily. Motif6 was specifically present in the *PavTCP7* and *PavTCP8* sequences of the CYC/TB1 subfamily, and the three PavTCP proteins of this subfamily displayed less similarity in motif composition and distribution than the other subfamilies. Despite PavTCP protein sequences in all three PavTCP subfamilies containing Motif9, it is noteworthy that the CIN subfamily contained multiple tandem Motif9—specifically 5 in *PavTCP5* and *3* in *PavTCP1*.

**Fig. 5:**
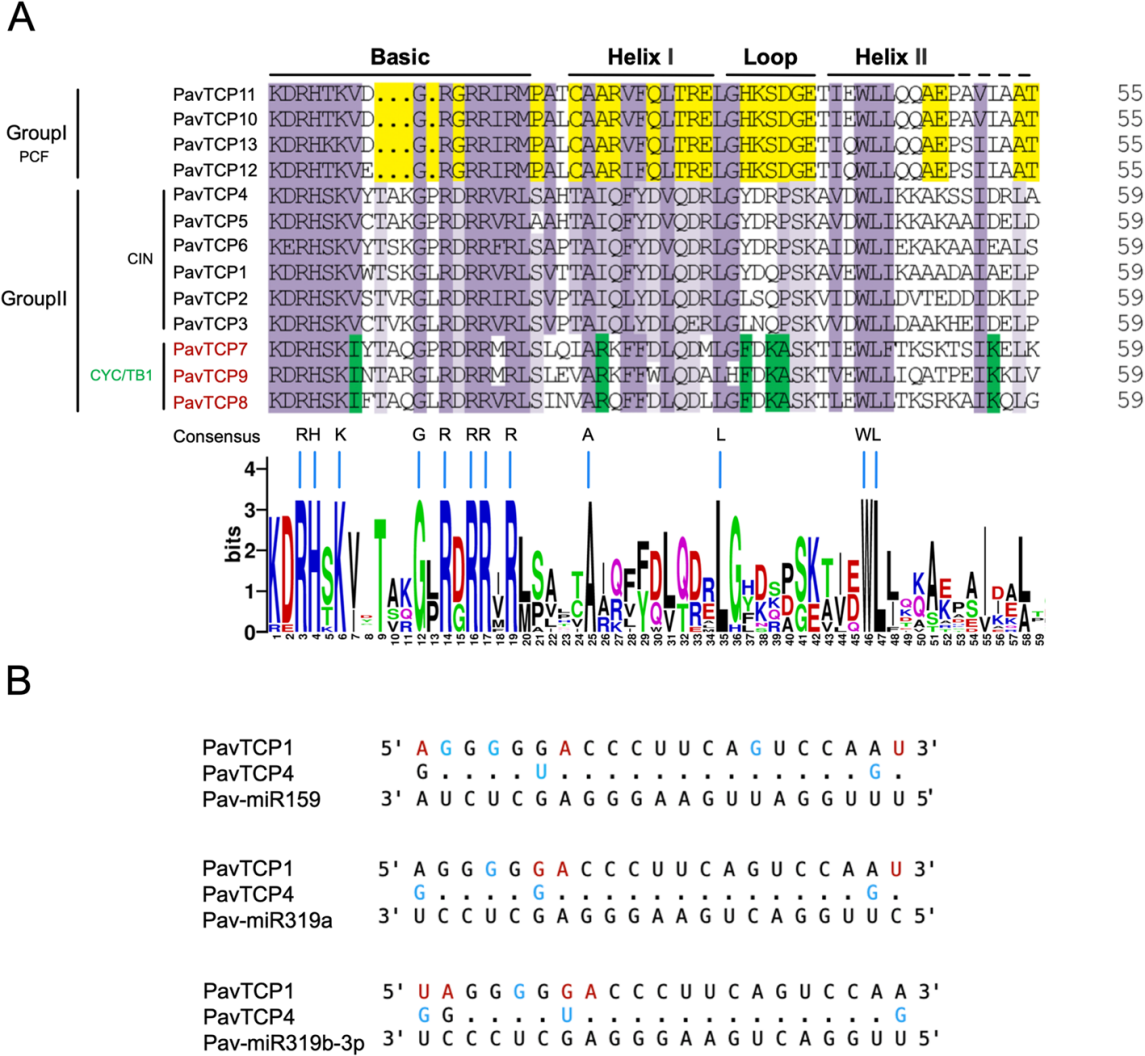
Multiple sequence alignment of sweet cherry TCP amino acid sequences. **A** Multiple sequence alignment and protein sequence logo of the TCP domain. The height of the stack suggested the sequence preservation at that position. Purple boxes showed the conserved amino acids in two TCP subfamilies; yellow, conserved in PCF; lilac, conserved in CIN; and green, conserved in CYC/TB1 clade. **B** Pav-miR319a, Pav-miR159, and Pav-miR319b-3p aligned to sweet cherry *TCP* genes. Mismatch and G-U wobbles are indicated in red and blue, respectively

The sweet cherry TCP structural domain consists of either 55 or 59 amino acids. According to the search results (Fig. 5A), we identified four conserved motifs: basic, helix I, loop, and helix II. Compared with Group II, the Group I class of PavTCP proteins contained four amino acid deletions in the basic motif region, which effectively distinguished the two classes of proteins. The motif composition of PavTCP proteins within the same subfamily was highly similar.

Additionally, we discovered that two genes from the CIN subfamily, *PavTCP1* and *PavTCP4*, contained putative Pav-miR159, Pav-miR139a, and Pav-miR139b-3p target sites (Fig. 5B). *PavTCP4* was associated with three Pav-miRNA oscillations, and mismatches were lower than those of *PavTCP1* Previous studies have shown that the CIN-type *TCP* genes *AtTCP2*–4 and *AtTCP10* in *Arabidopsis thaliana* undergo posttranscriptional regulation by miR319 [37], although in our study both genes partially mismatched with Pav-miRNA, post-transcriptional regulation of *PavTCP1* and *PavTCP4* by Pav-miR159, Pav-miR139a, and Pav-miR139b-3p is still possible.

### Prediction of protein interaction networks

**Fig. 6:**
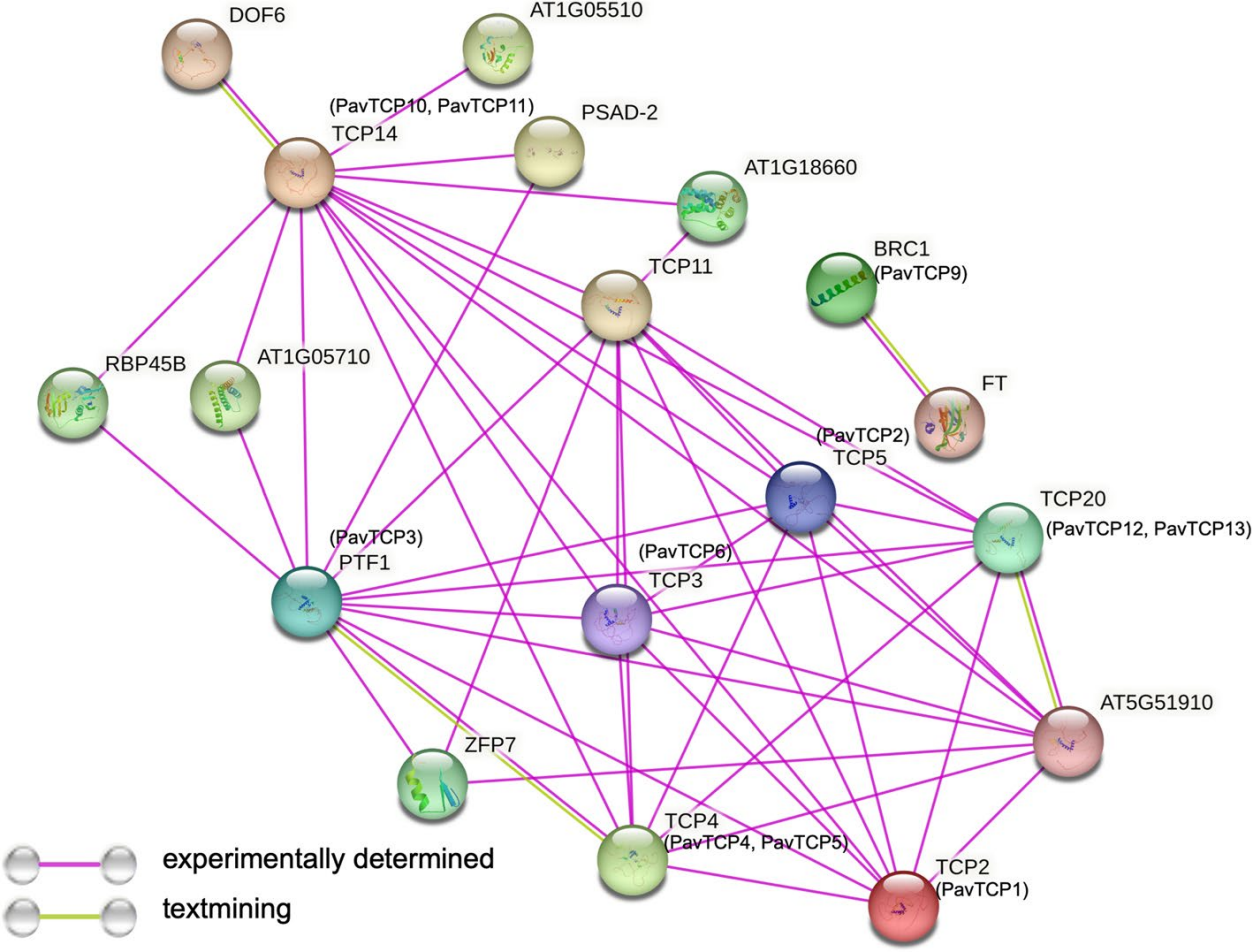
Protein interaction network. Protein interaction network based on Arabidopsis homologs of PavTCPs. Abbreviated names are genes reported in Arabidopsis. PavTCP proteins homologous to Arabidopsis are shown in parentheses

We first performed sequence comparisons using the STRING website to screen for Arabidopsis TCPs homologous to PavTCPs. The most likely homologous Arabidopsis TCPs were selected by combining the values of identity, bitscore, and e-value. However, considering the structural differences between Arabidopsis TCPs and PavTCPs, only preliminary predictions could be made in this step. The results suggest that homology between the 11 PavTCP proteins and known Arabidopsis proteins may exist. Protein interaction network analysis was then performed using Arabidopsis TCP proteins that may be homologous to sweet cherry (Fig. 6, Table S1). Most TCP proteins had close interactions with other proteins. Apart from BRC1, these proteins had eight or more protein interactions. PavTCP1, which may be homologous to AtTCP2, interacts with AtTCP4 (PavTCP4, PavTCP5) and PTF1 (PavTCP3) proteins. We found that some of the identified TCP proteins play significant roles in the light responses, and it has been demonstrated in previous studies that Arabidopsis TCP2 and PTF1 play important roles in light responses.

### Shading of sweet cherry fruit

**Fig. 7:**
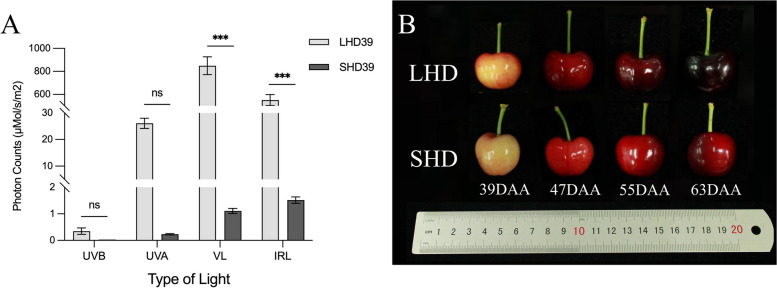
Bagging had a shading effect on the fruit. **A** Histogram of photon count statistics for each light quality inside and outside the bag after shading; **B** Phenotypic plots of sweet cherry fruits after shading at 39, 47, 55, and 63 days after anthesis. UVB indicates ultraviolet radiation b; UVA, ultraviolet radiation a; VL, visible light; IRL, infrared light

To examine the potential role of PavTCPs in sweet cherry fruit growth and development and light responses, we treated sweet cherry fruit with bag shading and collected samples from four periods with significant phenotypic differences in fruit. The photon counts in each spectral band inside the bag were markedly lower than those outside the bag (Fig. 7A). The total photon counts inside the bag were 0.1% of those in natural light, implying a shading rate of 99.9%. In other words, bagging had a profound shading effect on the sweet cherry fruits. Phenotypically, the fruit color was noticeably lighter after shading, and red deposition was reduced (Fig. 7B).

### Changes in various physiological indicators of sweet cherry after shading

**Fig. 8:**
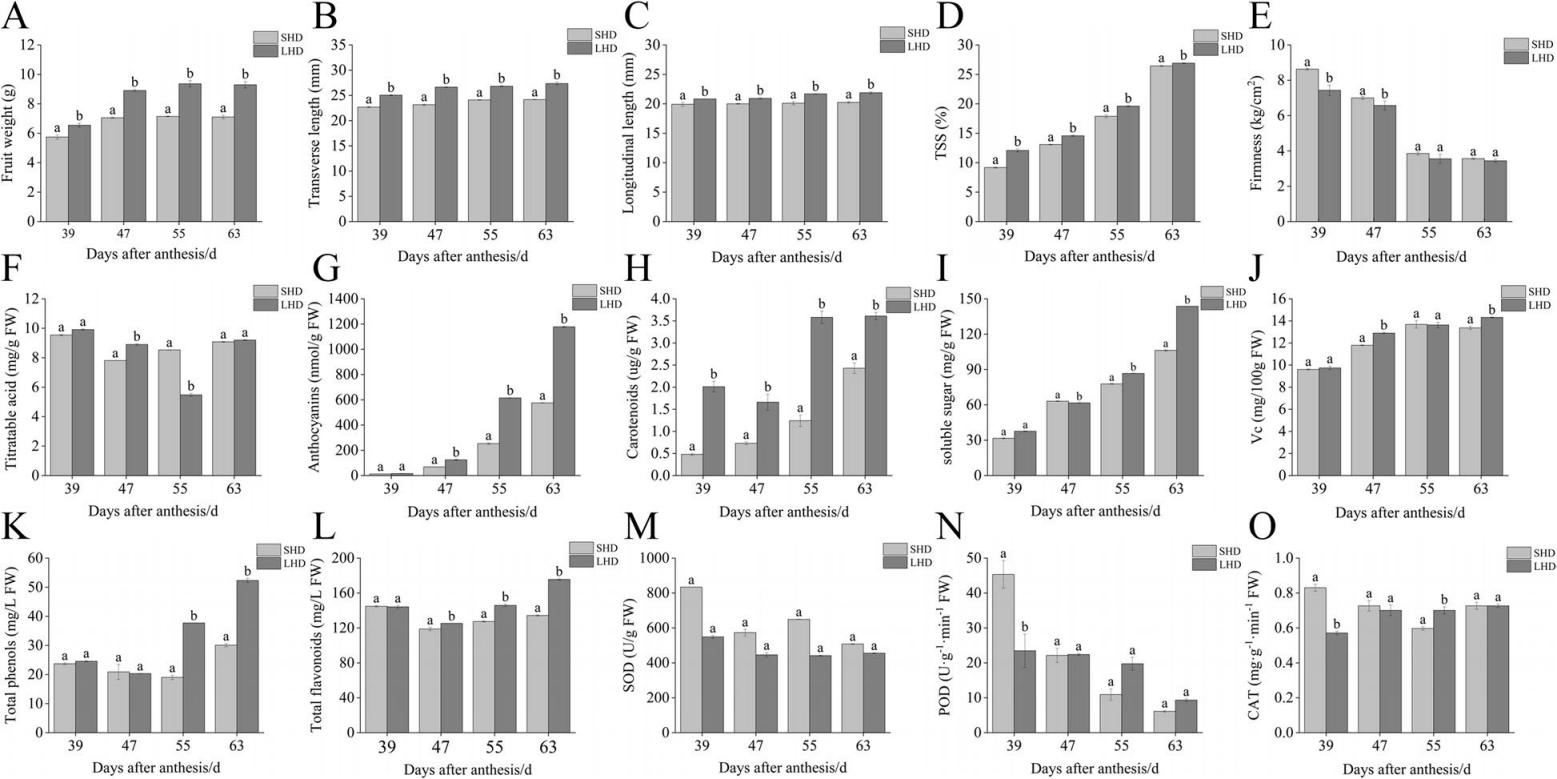
Changes in physiological indices of fruit after shading. **A** fruit weight per fruit; **B** fruit transverse length; **C** fruit longitudinal length; **D** soluble solids content; **E** firmness; **F** titratable acid; **G** anthocyanins; **H** carotenoids; **I** soluble sugars; **J** vitamin C (Vc); **K** total phenols; **L** total flavonoids; **M** superoxide dismutase (SOD) activity; **N** peroxidase (POD) activity; **O** catalase (CAT) activity

Subsequently, we measured a series of physiological indices of sweet cherry fruits (Fig. 8), including fresh fruit weight per fruit, fruit longitudinal and transverse diameter, soluble solids content, firmness, total anthocyanin content, carotenoid content, total flavonoid content, total phenol content, vitamin C content, soluble sugar content, titratable acid content, and activities of SOD, POD, and CAT enzymes. The results, as shown below, indicated that fruit firmness and SOD enzyme activity significantly increased at all four periods after shading. The titratable acid content of the fruit did not differ notably before and after the two periods, showing a decrease at 47 DAA and an increase at 55 DAA. Vitamin C content declined in the two periods after shading. POD and CAT enzyme activities increased significantly at 39 DAA, and then no significant difference was observed.

The remaining fruit weight, longitudinal and transverse meridians, soluble solids, total anthocyanins, carotenoids, total flavonoids, total phenols, and soluble sugar content were significantly $\textrm{decreased}$ after fruit shading. The differences in all the remaining indices, apart from the weight and longitudinal and transverse meridians of single fruits, were most pronounced 63 DAA This indicates that shading had the greatest effect on sweet cherry fruits 63 DAA.

### Correlation analysis of PavTCPs with physiological indicators

**Fig. 9:**
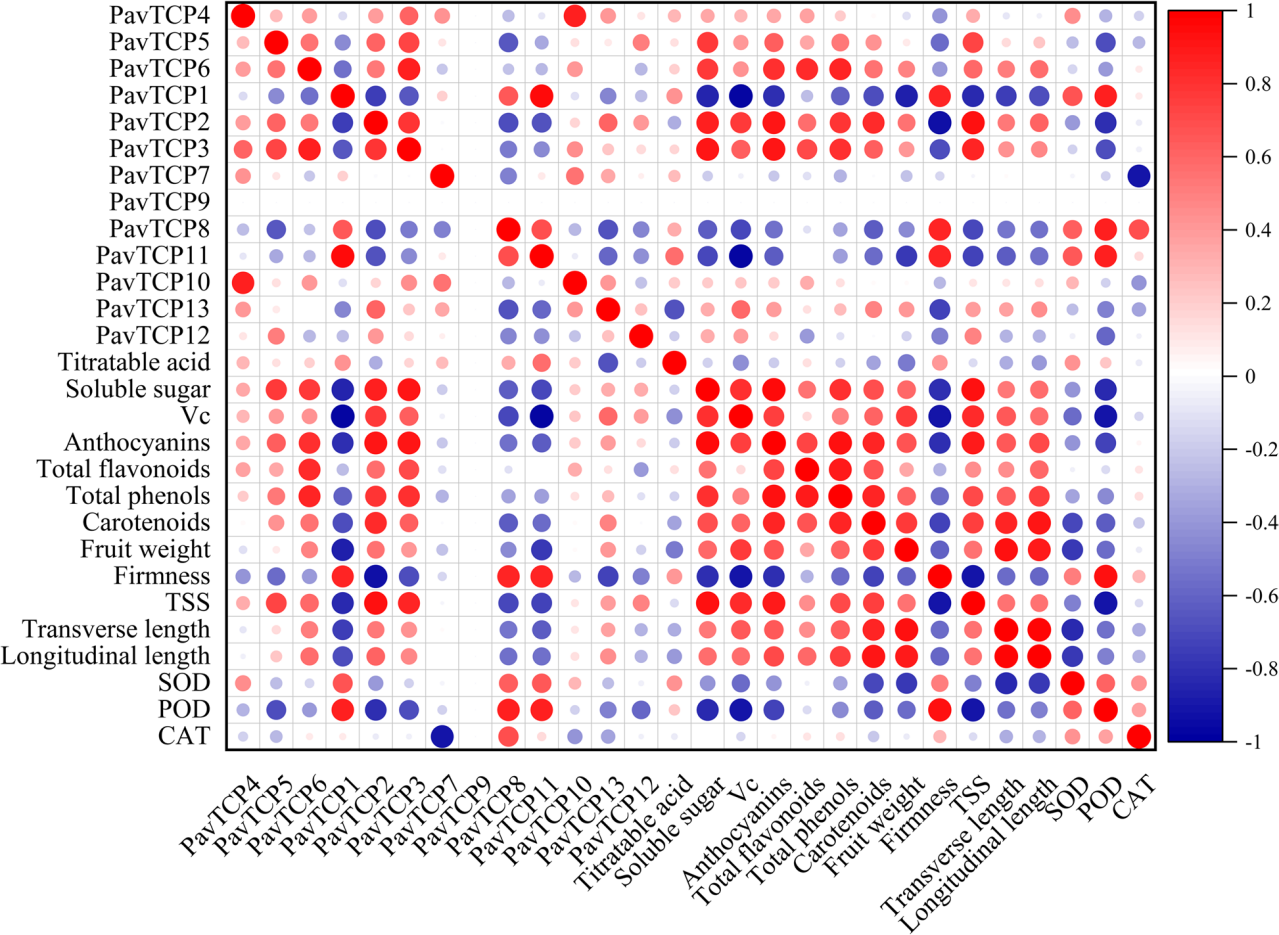
Heatmap of correlations between PavTCPs and 15 physiological indicators. The color and diameter of the circles in the graph represent the size of the correlation, red (1) represents a positive correlation, blue (− 1) represents a negative correlation, and the larger diameter of the circle represents a stronger correlation

We then performed correlation and PCA analyses of the average FPKM (Fragments Per Kilobase of exon model per Million mapped fragments) values of 13 PavTCPs genes (Table S2) with 15 physiological indices (Fig. 9, Table S3) at different times. The results indicated that the expression level of most members of the sweet cherry TCP transcription factor family had a strong correlation with the content of total anthocyanin, soluble sugar, and soluble solids contents of sweet cherry during fruit growth and development. The expression levels of most PavTCPs did not significantly correlate with the external quality of fruit (fruit weight per fruit, fruit longitudinal diameter, fruit transverse diameter), as well as the activities of fruit antioxidant enzymes (SOD, POD, CAT). Interestingly, the correlation between POD and most PavTCPs was high and significant. For instance, the correlation between POD and *PavTCP1*, *PavTCP8*, and *PavTCP11* was 0.86, 0.87, and 0.86, respectively, and the correlation was highly significant. However, a significant negative correlation (− 0.81) was observed between POD and *PavTCP2*. Among the PavTCPs, *PavTCP2*, *PavTCP3*, and *PavTCP6* had significant positive correlations with the total anthocyanin, soluble sugar, and soluble solid contents of sweet cherries. Conversely, *PavTCP1* exhibited a significant negative correlation with these indices (correlation coefficient < − 0.8, Table S3). These results suggest that *PavTCP1*, *PavTCP2*, *PavTCP3*, and *PavTCP6* may play opposing regulatory roles in sweet cherry growth and development.

### Q-PCR validation

**Fig. 10:**
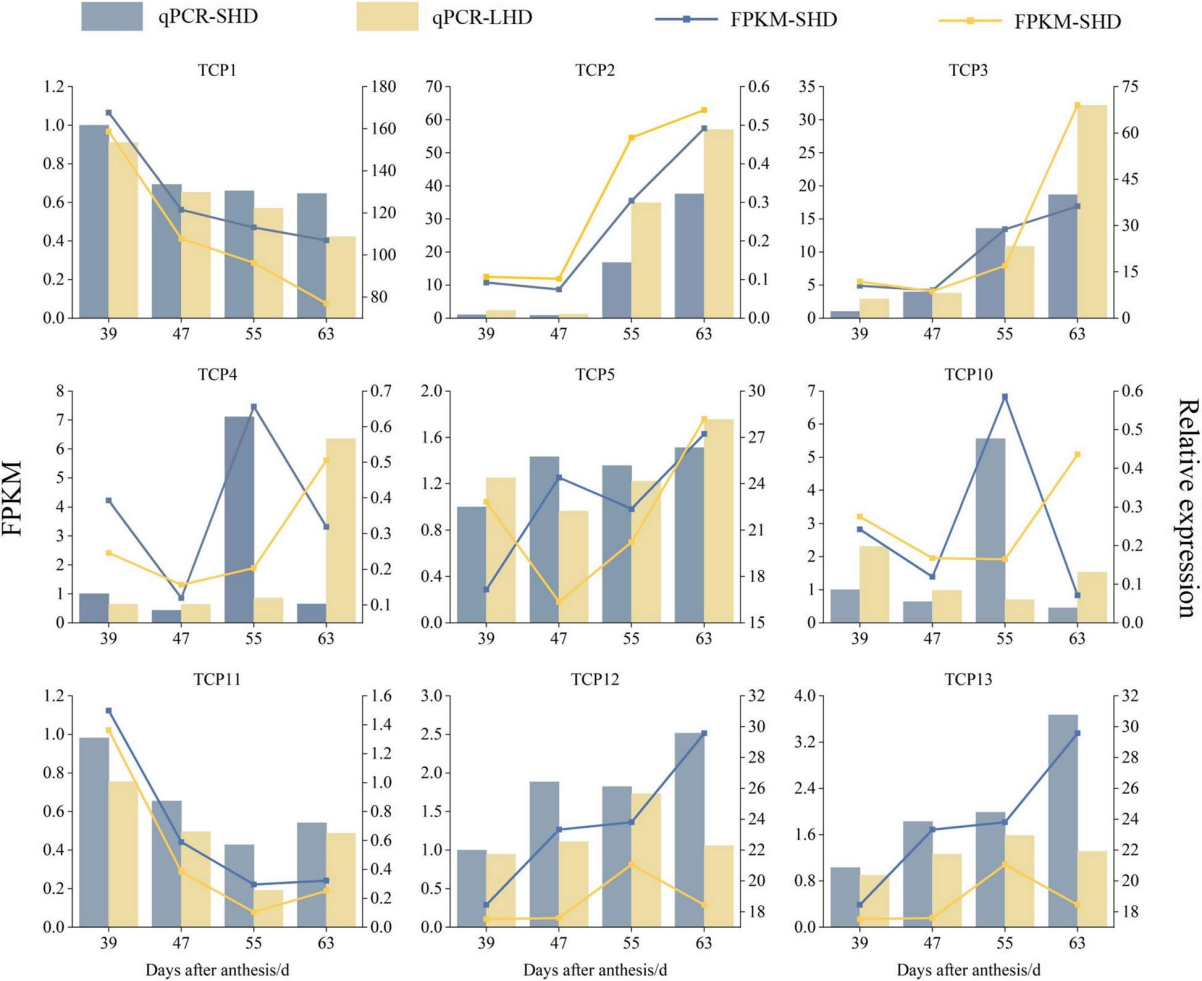
qRT–PCR validation of the *PavTCPs* after shade treatment

To validate the transcriptome data, we performed q-PCR analysis on 13 members of the sweet cherry *TCP* transcription factor family. *PavTCP6*, *7*, *8*, and *9*, which had extremely low FPKM values (refer to Table S2 for FPKM values of all PavTCPs), returned inconclusive q-PCR results. The q-PCR results for the remaining 9 PavTCPs (Fig. 10) showed that the trends of expression changes detected by both methods were similar over the 4 periods (refer to Table S4 for standard deviation of FPKM and q-PCR values). Pearson’s correlation coefficient (S5) showed that the correlation of the relative quantitative qPCR values of the samples with the FPKM values ranged from 0.871 (*PavTCP11*) to 0.989 (*PavTCP1*), which increased the reliability of the transcriptome data.

## Discussion

The *TCP* family is a plant-specific family of transcription factors that primarily regulate flower symmetry, axillary bud apical growth, and bud dormancy in plants. In this study, we identified a total of 13 PavTCPs from sweet cherry fruits, which belonged to three *TCP* subfamilies. Compared with other Rosaceae, PavTCPs have fewer members. For example, the apple (*Malus domestica*) *TCP* family has four times as many members as PavTCPs [38], and the pear (*Pyrus bretschneideri*) *TCP* family is 40.3 times larger than PavTCPs [38], while the *TCP* family of *P. mume has 1*.46 times more *TCP* $\textrm{members}$ than PavTCPs [39]. Intraspecific and interspecific colinearity analyses revealed that repetitive events contributed more to the expansion of the *PavTCP* gene family, and the sweet cherry *TCP* transcription factor family was more closely related to *MdTCP* during development. Among the conserved motifs and gene structures, all three subfamilies of the sweet cherry *TCP* gene family contained specific motifs, and the UTR-CDS structures of the PavTCPs displayed high simplicity. Cis-acting element analysis showed that members of the sweet cherry *TCP* transcription factor family contain a large number of elements related to hormones and light responses, suggesting potentially important roles in sweet cherry growth and development.

miRNAs are small non-coding RNAs that regulate gene expression by recognizing homologous sequences and interfering with transcription, translation, or epigenetic processes [40]. miR319 and miR159, although encoded by different genes and precursors, are highly similar in terms of their members [41]. In this study, we discovered that two genes from the CIN subfamily, *PavTCP1* and *PavTCP4*, contain putative target sites for Pav-miR159, Pav-miR319a, and Pav-miR319b-3p. A similar occurrence was found in eggplant (*Solanum melongena*), as reported by Li et al., who suggested that three miRNAs (miR319a, miR319b, and miR319c-3p) might be the major regulators targeting *SmTCP* [42]. *TCP4*, a member of the class II *TCP* subfamily, is regulated by miR319 and can directly inhibit cell proliferation in Arabidopsis leaves [43]. These findings suggest that the potential post-transcriptional regulation of *PavTCP1* and *PavTCP4* by Pav-miR159, Pav-miR319a, and Pav-miR319b-3p, despite partial mismatching of both *PavTCP1* and *PavTCP4* with Pav-miRNAs in sweet cherry.

The protein-protein interaction network of a specific gene family provides information about the relationships among family members. After rigorous screening, we obtained the TCP proteins in Arabidopsis that are most likely to be homologous to sweet cherry. In addition to the common functions of the TCP family, such as involvement in ovule development [44], some of the identified proteins, such as TCP2 (PavTCP1) and PTF1 (PavTCP3), also play significant roles in the light response. TCP2, a member of the CIN subfamily of AtTCPs, is known to positively regulate light responses by promoting light-regulated transcription of genes downstream of the CRY1 signaling pathway, such as CAB, CHS, HY5, and HYH [45]. Similarly, Arabidopsis PTF1, which may be homologous to PavTCP3, has also been reported to be associated with light responses [46]. These observations suggest that TCPs may play a crucial role in light responses in plants. Given the potential structural differences between Arabidopsis TCPs and PavTCPs, we only tentatively hypothesize here that PavTCPs, which are highly homologous to Arabidopsis TCPs, may also have similar functions.

Light serves not only as the energy source for plant photosynthesis but also plays a role in transmitting information [47]. It controls various light responses throughout the plant’s growth cycle, including seed germination, phototropic growth, flowering, color accumulation, and aging [48]. Based on the results of cis-acting elements and protein-protein interaction network predictions, to further investigate the role of PavTCPs in sweet cherry fruit growth, development, and light responses, we shaded sweet cherry fruits by bagging and measured 15 common fruit physiological indices. After shading, sweet cherry fruits decreased in size, and the single fruit weight decreased, mirroring the findings of previous research [49]. Furthermore, fruit appearance, color, and anthocyanin content were significantly altered, with the latter greatly reduced, as previously reported in apple [50], pear [51], grape [52], and other fruits. Sugar, an important precursor substance for anthocyanin biosynthesis [53], was also suppressed after fruit shading. Vitamin C, a critical nutrient for humans and a significant indicator of fruit quality [54] was likewise less accumulated in shaded sweet cherries. Other aspects, such as total phenols, total flavonoids, titratable acid, SOD enzyme activity, and CAT enzyme activity, also exhibited varying degrees of reduction post-shading. Conversely, fruit firmness increased after shading, similar to findings by Muhammad [55]. POD enzyme activity also showed a minor increase post-shading. We then correlated the 13 PavTCPs with 15 physiological indices of sweet cherry fruit, demonstrating that total anthocyanin content, soluble sugar content, and soluble solids content had significant correlations with most members of the sweet cherry *TCP* transcription factor family.

Anthocyanin is a type of water-soluble pigment in the flavonoid family [56]. It not only provides bright colors to plants, attracting birds and insects for pollination and reproduction but also plays a significant role in protecting human eyesight and preventing cardiovascular diseases [32, 57]. The primary anthocyanin species in ‘Hong Deng’ sweet cherries is cyanidin 3-O-rutinoside [58], which is a vital source of the cherries’ red color and significantly influences consumers’ purchasing desire. Studies have shown that *TCP* transcription factors regulate anthocyanin biosynthesis in some plants, a role correlated with light intensity. For instance, in apple, the induction of anthocyanin biosynthesis by different light intensities relies heavily on the function of *MdMYB1* and the class II *TCP* transcription factor *MdTCP46* [59]. Apple callus overexpressing *MdTCP46* exhibited a substantial increase in anthocyanin content at high light intensities [60]. This transcription factor also interacts with *MdBT2*, a BTB protein responsive to high light intensity, to regulate anthocyanin biosynthesis [60]. In *Arabidopsis thaliana*, a *TCP* transcription factor (class I *TCP* transcription factor *TCP15*) regulates anthocyanin accumulation under varying light intensities, but this transcription factor is negatively regulated [22]. The class II *TCP* transcription factor *FvTCP9* in strawberry positively regulates anthocyanin accumulation by interacting with *FaMYC1* [61, 62]. Similarly, the class II *TCP* transcription factor *MhTCP4* in *Malus halliana* also positively regulates anthocyanin accumulation [9]. This aligns with earlier findings suggesting that class I and class II *TCP* transcription factors have antagonistic functions [63]. In our study, the class II *TCP* transcription factors *PavTCP2* and *PavTCP3* correlated significantly positively with anthocyanin content, indicating that these transcription factors may positively regulate anthocyanin biosynthesis in sweet cherry, which is consistent with the results of previous studies. PavTCP2 exhibited a significant negative correlation with POD and a highly significant positive correlation with soluble sugars, anthocyanins, and TSS. This suggests that PavTCP2 may promote the accumulation of anthocyanins by inhibiting the activity of POD. Similarly, the study conducted by Rehman et al. also indicates that an increase in POD activity results in the degradation of anthocyanins in fruit [64]. Intriguingly, the *TCP* transcription factor *PavTCP1*, also from the class II subfamily, was significantly negatively correlated with anthocyanin content, suggesting that *TCP* transcription factors of the same subfamily may also exhibit antagonistic functions.

Sugars are the precursors for anthocyanin synthesis, and most soluble solids are soluble sugars. Past reports have indicated that the class I *TCP* transcription factor *PvPCF5* affects the soluble sugar content in switchgrass [26]. In tomato, transcriptomic data suggested that the fruit’s soluble sugar content might also be influenced by the *TCP* transcription factor [65]. In our study, the fruit’s soluble sugar content and soluble solids content were significantly correlated with most of the PavTCPs. And the pattern of these correlation was similar to that of anthocyanin content—significantly positively correlated with *PavTCP2* and *PavTCP3* and negatively correlated with *PavTCP1*. These reports and experimental findings suggest that *TCP* transcription factors may also have regulatory roles in plant sugar metabolism pathways.

Various studies have indicated that the mRNA abundance of *TCP* transcription factors can be regulated post-transcriptionally by miRNAs, subsequently affecting plant growth and development. For instance, in switchgrass, *PvPCF5* can directly bind to miR319 and regulate lignin biosynthesis [26]. In watermelon, *TCP* transcription factors may also be regulated by miR319 to enhance fruit salt tolerance [27]. Similarly, in the model plant *Arabidopsis*, hormone jasmonate synthesis, cell proliferation, and petal development are regulated by miR319-mediated *TCP* transcription factors [43, 66, 67]. Mac-miR159, which targets the *TCP* gene in banana (*Musa acuminata*), can be cold-induced [68]. In the sweet cherry *TCP* transcription factor family, both *PavTCP1* and *PavTCP4* of the CIN subfamily contain potential binding sites for miR159, miR319a, and miR319b-p. Given that *PavTCP4* had a low correlation with both fruit physiological indices in the correlation analysis, we hypothesized that the potential negative regulatory role of *PavTCP1* in anthocyanin, soluble sugar, and soluble solids content of fruit after shading was mediated by miR319a, miR319b-3p, or miR159.

## Conclusion

In this study, we identified a total of 13 members of the sweet cherry *TCP* transcription factor family. Analysis of cis-acting elements revealed that PavTCPs contain a substantial number of light-responsive elements. Further analyses of miRNA and protein interactions demonstrated that *PavTCP1* and *PavTCP4* contain putative target sites for Pav-miR159, Pav-miR319a, and Pav-miR319b-3p and that homologous proteins in Arabidopsis of *PavTCP1* and *PavTCP3* play a crucial role in light responses. Through correlation analysis with physiological indices of sweet cherry fruits at four stages post-shading, we found that *PavTCP1*, *PavTCP2*, and *PavTCP3* were significantly correlated with total anthocyanins, soluble sugars, and soluble solids in sweet cherry. Consequently, these genes may play vital roles in the light responses of sweet cherry fruits, with the role of *PavTCP1* potentially mediated by Pav-miR159, Pav-miR319a, and Pav-miR319b-3p. However, the regulatory network of plant growth and development is intricate and complex, the specific roles of *TCP* transcription factors in the growth and development of sweet cherry fruit should be investigated further.

## Materials and methods

### Plant materials and treatment

We chose sweet cherry (*Prunus avium* L.) fruits which were grafted onto *Prunus tomentosa* as the experiment materials. We got permission to do the experiment at Buwa Village experimental base in Weizhou Town, Wenchuan County, Aba Tibetan and Qiang Autonomous Prefecture, Sichuan Province, China. Six fruit trees with consistent growth potential were randomly selected and divided into two plots, with three trees each for replication. The experiment involved shading bagging treatment for ‘Hong Deng’ sweet cherry fruits (SHD), with normal light fruits without bagging as the control (LHD). Bagging was carried out for sweet cherry fruits in the treatment group plot on April 25, 2022 (23 days after anthesis, 23 DAA). Sampling started on 39 DAA, and sweet cherry fruits of uniform size, pest- and disease-free, and without mechanical damage were collected from the canopy periphery of each tree in each plot in the east, south, west, and north directions using a random sampling method. This was repeated every 7 days until fruit ripening (63 DAA), totaling four collection periods (39 DAA, 47 DAA, 55 DAA, and 63 DAA). Each sample was immediately placed in an icebox and transported to the laboratory for fresh sample physiological index determination. Then, the samples were swiftly cut into uniform blocks, flash-frozen in liquid nitrogen, wrapped in tinfoil, and stored in a − 80 °C refrigerator. Each sample had three biological replicates.

### Determination of spectral data and physiological indicators in fruit bags

An AvaSpec-ULS2048L multipurpose fiber optic spectrometer was used to determine the spectra inside the fruit bag. The single fruit weight of sweet cherries was determined using an analytical balance, while the longitudinal and transverse diameters of fruits were measured using digital Vernier calipers. The GY-1 firmness tester was employed to determine fruit firmness, and a MASTER-M handheld sugar meter was used to ascertain the soluble solids content in fruits. The total anthocyanin content in fruits was determined by the 1% hydrochloric acid-methanol method [69]. The anthrone colorimetric method was used to assess the content of soluble sugars [70], while the acid–base titrimetric method was used to evaluate the titratable acid content [71]. The vitamin C content was ascertained by Rey’s method [72], while total flavonoids and total phenols were determined using Daniela’s method [73]. Carotenoid content was established using Carvalho’s method [74], and the activities of superoxide dismutase (SOD), peroxidase (POD), and catalase (CAT) enzymes were determined using ELISA kits (enzyme immunoassay, Yancheng, China).

### Identification and analysis of TCP gene family members

#### Search and identification of sweet cherry TCP gene family members

The TCP family module sequence (PF03634) and sweet cherry protein sequence information were downloaded from the Pfam database (http://pfam.xfam.org/) and GDR (https://www.rosaceae.org, The version we used was *Prunus avium* Genome v1.0.a1), respectively. The TCP protein sequence was then retrieved from the sweet cherry protein sequence using HMMER software. The candidate proteins were further submitted to the NCBI-CDD and Pfam platforms for structural domain confirmation. Physicochemical properties, such as protein molecular weight and isoelectric point, were analyzed for the confirmed TCP protein sequences using the ExPASy website (http://web.expasy.org/protparam/), and WOLF PSORT (http://www.genscript.com/wolf-psort.html) was used for subcellular localization analysis. The *TCP* gene sequences of Arabidopsis were downloaded from the TAIR (https://www.arabidopsis.org/) website, and the *TCP* gene sequences of *Malus domestica* Borkh. were downloaded from NCBI. The obtained sweet cherry *TCP* gene family members were subjected to amino acid multiple sequence comparison using DNAMAN software.

#### Phylogenetic analysis

All TCP protein sequences of *Prunus avium* L。, *Arabidopsis thaliana*, and *Malus domestica* Borkh. were subjected to multiple sequence alignment using Clustal X software. The phylogenetic analysis was later performed using the neighbor-joining method using MEGA software with the calibration parameter bootstrap repeated 1000 times to construct the phylogenetic tree. The phylogenetic trees were landscaped using the online website Evolview (http://www.evolgenius.info/evolview/#/login).

#### Chromosome distribution and inter- and intra-genomic homologous sequence analysis

We downloaded the sequence information of Apple and Arabidopsis to analyze the collinearity. Intraspecific and interspecific collinearity relationships of the PavTCP gene family were analyzed using MCScanX and visualized using TBtools software (https://github.com/CJ-Chen/ TBtools).

#### Conserved domains and motif analysis

By logging into the GSDS server (http://gsds.cbi.pku.edu.cn/), the structural patterns of introns and exons of *TCP* family genes were mapped by comparing the coding sequences with gene sequence information. The conserved motifs of sweet cherry *TCP* family gene members were analyzed using MEME online tool (http://mememe-suite.org/), and the size of the conserved motif was set to be between 10 and 100 amino acids, with the maximum number of output structural domains set to 10.

The cis-acting element prediction analysis was performed with a base sequence 2000 bp upstream of the sweet cherry *PavTCP* gene family start codon using the Plant CARE (http://bioinformatics.psb.ugent.be/webtools/plantcare/html/) online software. Finally, the results of the above analyses were visualized using TBtools software.

#### Chromosomal localization and miR159/miR319 target site prediction

The chromosomal location and structural information of *TCP* family gene members were obtained from the downloaded sweet cherry genome. The chromosomal location distribution of *TCP* family genes was mapped using TBtools software. To predict miR159 and miR319 target sites, the nucleotide sequences of PavTCP were analyzed using the psRNATarget (https://www.zhaolab.org/psRNATarget/) online website. The sequences of Pav-miR319a, Pav-miR159, and Pav-miR319b-3p are available in Table S6.

#### Prediction of protein interaction networks of sweet cherry TCP gene family members

Sweet cherry PavTCP protein–protein interactions were analyzed using the STRING website (https://cn.string-db.org), with Arabidopsis chosen as the reference organism. After performing blast analysis, the highest scoring Arabidopsis homologous gene (Bitscore) was used to construct the network. Genes that did not interact with other genes were removed.

### qRT–PCR analysis

The transcriptome data and the whole experiment used in this study have been published on the National Center for Bioinformation website (https://ngdc.cncb.ac.cn/gsa/browse/CRA011359). We first downloaded the transcriptome data from the National Center for Bioinformation website and then compared it to the reference genome using HISAT2. The number of reads for each transcript was counted using the featureCounts tool in the subread software. And then the FPKM value of each gene was calculated. Total RNA was extracted using the Plant Total RNA Extraction Kit. cDNA was synthesized immediately after using the HiScript® III RT SuperMix for qPCR (+gDNA wiper) kit. cDNA was synthesized by reverse transcription PCR under the following conditions: 25 °C for 10 min, 50 °C for 15 min, and 85 °C for 5 min. The first strand of cDNA synthesized by reverse transcription was stored at − 20 °C for spare parts. qRT–PCR was performed using the CFX96TM Real-Time System (Bio-Rad, California, USA) and 2 × TSINGKE® Master qPCR Mix (SYBR Green I) (TSINGKE, Beijing, China) reagents. Primer 6.0 was used to design PCR primers (Table S5). The cDNA was used as the template, and the β-actin gene was used as the internal reference to analyze the expression of related genes by real-time fluorescence quantitative PCR. Three biological and technical replicates were performed for each reaction, and the amplification program was as follows: predenaturation at 95 °C for 30 s; denaturation at 95 °C for 10 s, annealing and extension at 60 °C for 30 s, with 40 cycles; the relative expression of the genes was calculated using 2^-ΔΔCt^ [75].

### Data processing and analysis

Three sets of replicates were determined for each indicator in each period. The data were sorted, checked, and plotted using Excel, SPSS 27.0, and GraphPad Prism 9.

### Supplementary Information

**Additional file 1.**

**Additional file 2.**

## Data Availability

The transcriptome proposed in the study are deposited in the National Genomics Data Center database. You can query transcriptome data by visiting thelink (https://ngdc.cncb.ac.cn/gsa/browse/CRA011359) (BioProject: PRJCA010046; accession number: CRA011359).
