# Supplementary material for: Sweet cherry TCP gene family analysis reveals potential functions of PavTCP1, PavTCP2 and PavTCP3 in fruit light responses
Source: BMC Genomics. 2024 Jan 2;25:3. doi: 10.1186/s12864-023-09923-z (PMC10759647; doi:10.1186/s12864-023-09923-z)
Supplement: Supplementary file 1 — Additional file 1. [file 12864_2023_9923_MOESM1_ESM.docx]

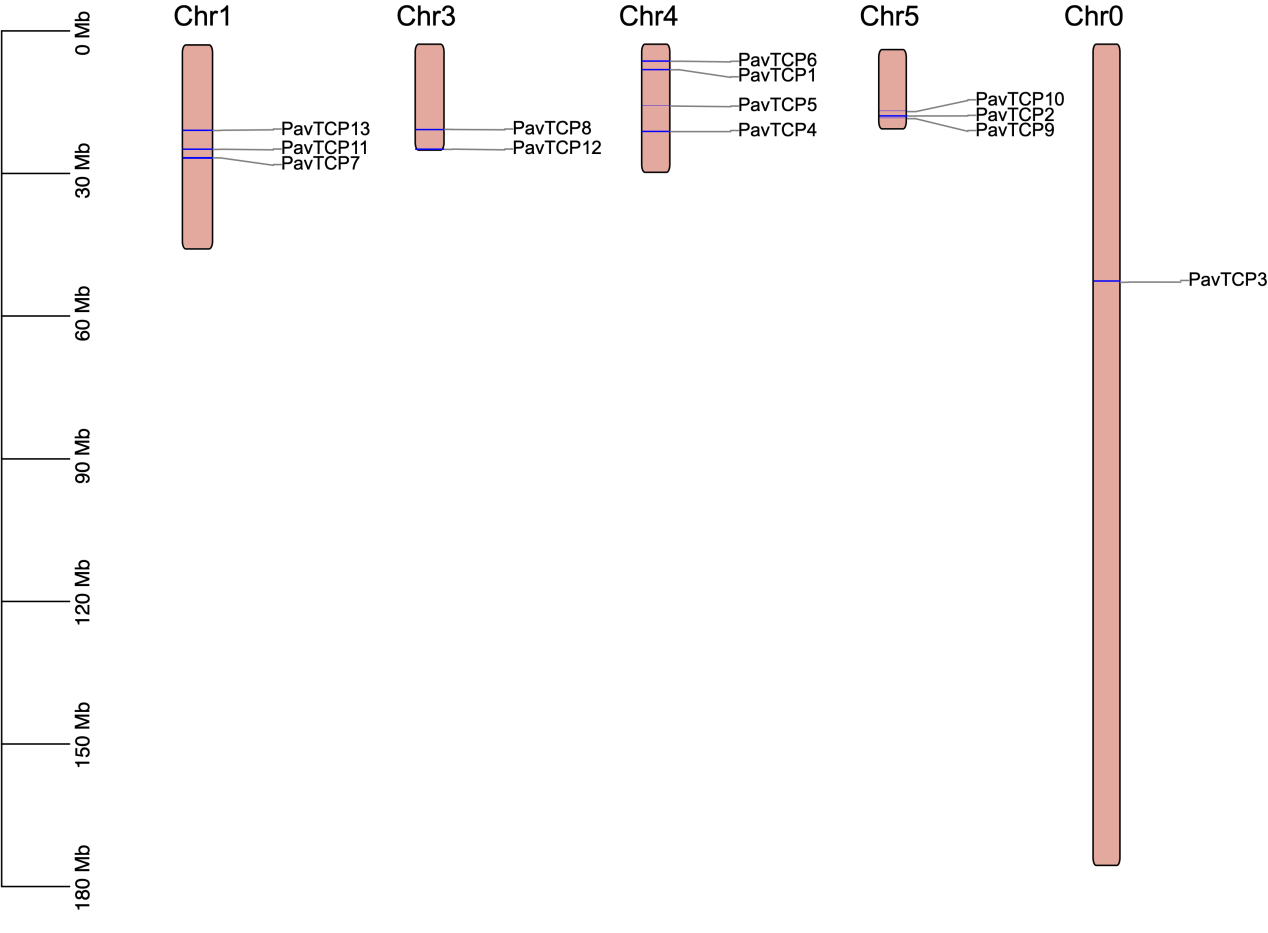


Fig. S1 Distribution of PavTCPs on chromosomes.


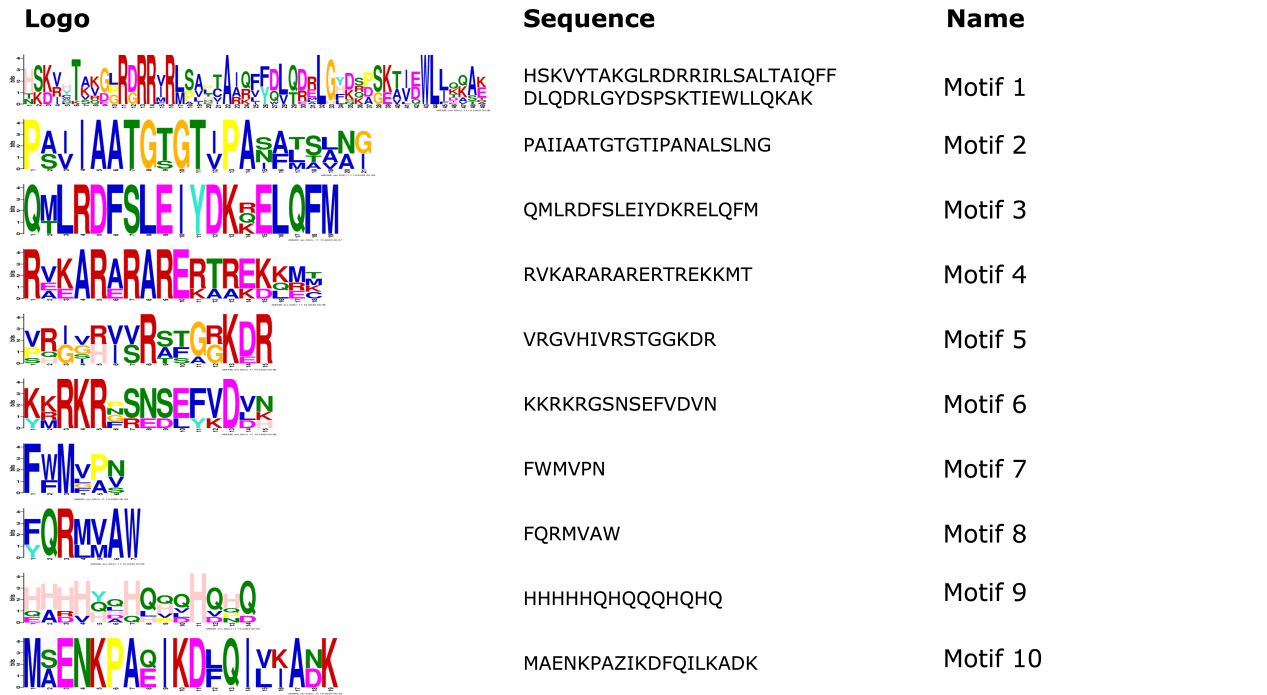


Fig. S2 Sequence logo of conserved domains found in PavTCP family. The X-axis shows the conserved sequences of the domains. The coservation of residues are indicated by the height of the letters. The Y-axis represents the conservation of the amino acid.


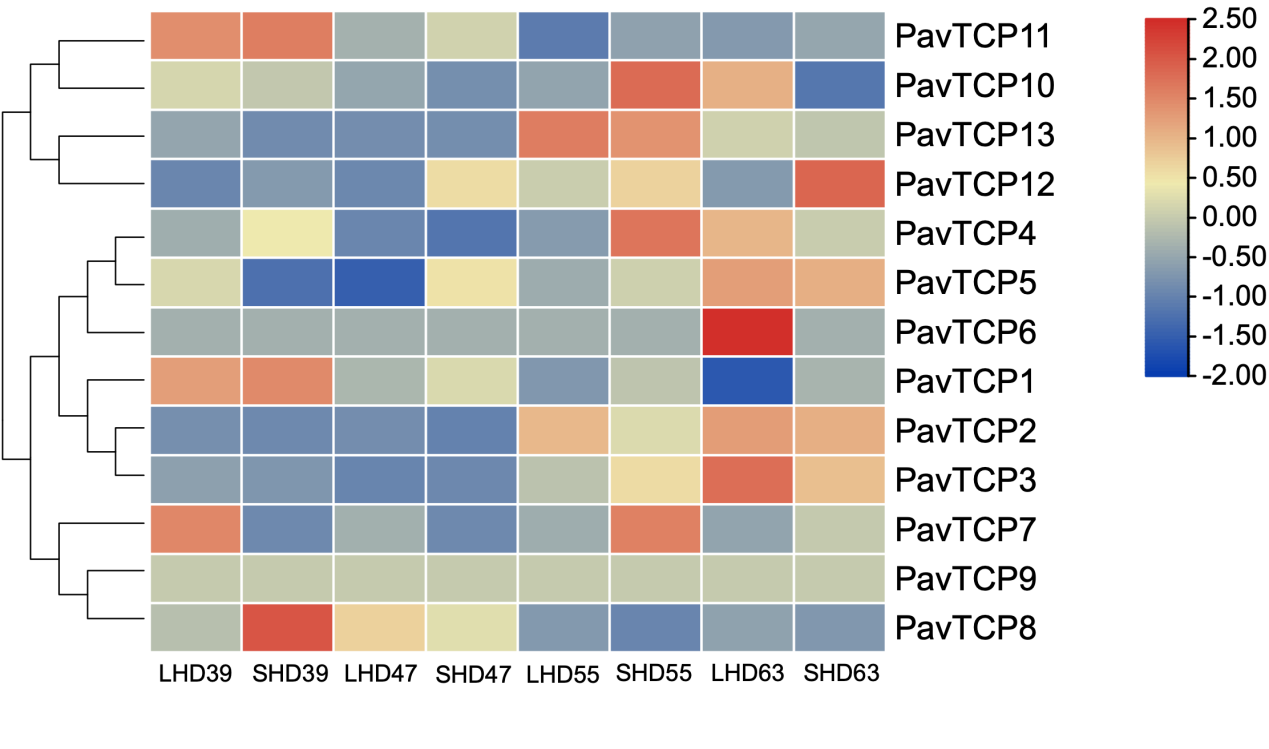


Fig. S3 Expression level heat map of 13 PavTCPs.
